# TRAIL regulatory receptors constrain human hepatic stellate cell apoptosis

**DOI:** 10.1038/s41598-017-05845-5

**Published:** 2017-07-17

**Authors:** Harsimran D. Singh, Itziar Otano, Krista Rombouts, Kasha P. Singh, Dimitra Peppa, Upkar S. Gill, Katrin Böttcher, Patrick T. F. Kennedy, Jude Oben, Massimo Pinzani, Henning Walczak, Giuseppe Fusai, William M. C. Rosenberg, Mala K. Maini

**Affiliations:** 1 Division of Infection and Immunity, UCL, London, UK; 2 Institute of Liver and Digestive Health, UCL, London, UK; 3 Centre for Cell Death, Cancer, and Inflammation, Cancer Institute, UCL, London, UK; 4 Monash University, Melbourne, Australia; 5 Department of Gastroenterology, Guy’s and St Thomas’ Hospital, London, UK; 6 Hepatology, Centre for Immunobiology, Blizard Institute, Barts and the London School of Medicine and Dentistry, QMUL, London, UK

## Abstract

The TRAIL pathway can mediate apoptosis of hepatic stellate cells to promote the resolution of liver fibrosis. However, TRAIL has the capacity to bind to regulatory receptors in addition to death-inducing receptors; their differential roles in liver fibrosis have not been investigated. Here we have dissected the contribution of regulatory TRAIL receptors to apoptosis resistance in primary human hepatic stellate cells (hHSC). hHSC isolated from healthy margins of liver resections from different donors expressed variable levels of TRAIL-R2/3/4 (but negligible TRAIL-R1) *ex vivo* and after activation. The apoptotic potential of TRAIL-R2 on hHSC was confirmed by lentiviral-mediated knockdown. A functional inhibitory role for TRAIL-R3/4 was revealed by shRNA knockdown and mAb blockade, showing that these regulatory receptors limit apoptosis of hHSC in response to both oligomerised TRAIL and NK cells. A close inverse *ex vivo* correlation between hHSC TRAIL-R4 expression and susceptibility to apoptosis underscored its central regulatory role. Our data provide the first demonstration of non-redundant functional roles for the regulatory TRAIL receptors (TRAIL-R3/4) in a physiological setting. The potential for these inhibitory TRAIL receptors to protect hHSC from apoptosis opens new avenues for prognostic and therapeutic approaches to the management of liver fibrosis.

## Introduction

Chronic liver inflammation and injury from a variety of insults trigger a dynamic, reversible wound-healing response, in which matrix deposition is accompanied by matrix degradation. If there is chronic or repetitive injury, persistent accumulation of extracellular matrix and insufficient tissue remodelling lead to the formation of scar tissue^1^. The resultant liver fibrosis can ultimately lead to cirrhosis, portal hypertension and liver failure, responsible for more than a million deaths annually worldwide^2^. Targeted therapies able to specifically halt the progression and/or promote regression of liver fibrosis are therefore urgently needed.

Hepatic stellate cells (HSC), liver-specific pericytes residing in the Space of Disse, are the main cellular mediators of fibrogenesis in the liver^1, 3, 4^. In the quiescent state they contain multiple retinoid-rich droplets; upon liver injury they are activated to differentiate into proliferative and contractile myofibroblast-like cells, that produce the extracellular matrix components of scar tissue^3^. Termination of their pro-fibrogenic activity requires that HSC undergo apoptosis, senescence or reversion to a quiescent state^4, 5^. Degradation of extracellular matrix will then outweigh new deposition, allowing fibrosis resolution and restoration of liver architecture^4, 5^.

It is increasingly recognised that many different components of the immune system have the capability to either promote or limit HSC activation and survival^3, 6, 7^. Amongst these, NK cells are of particular interest because of their striking enrichment within the human liver, including a large CXCR6^+^Tbet^hi^Eomes^lo^ liver-resident subset^8^. In animal models, depletion of NK cells results in severely accelerated fibrosis progression whereas their activation ameliorates it^9–11^, suggesting that they play a major role in limiting fibrogenesis. NK cells can interact with HSC through a number of receptor/ligand pairs and have been shown to be able to kill them in an NKG2D and TRAIL-dependent manner^10, 12–14^. We have previously shown activation of the TRAIL pathway in the HBV-infected liver; the ligand is induced on NK cells, allowing them to target hepatocytes and HBV-specific T cells, both of which upregulate the death-inducing receptor TRAIL-R2^15–17^.

In this work we have therefore focused on the potential for the TRAIL pathway to regulate stellate cell apoptosis. The ligand TRAIL has the capacity to initiate cell death by engagement with receptors TRAIL-R1 and TRAIL-R2, bearing intracellular death domains^18–21^. However, TRAIL can also bind to regulatory (decoy) cell-bound receptors TRAIL-R3 (DcR1) or TRAIL-4 (DcR2) that may protect against cell death, although to date there has been a scarcity of physiological demonstrations of this phenomena (demonstrable in over-expression systems^18, 22–25^). We have confirmed TRAIL-dependent killing of primary human HSC (hHSC) by using lentiviral-mediated shRNA knockdown of the death-inducing receptor TRAIL-R2. We found that hHSC also express TRAIL-R3 and TRAIL-R4, both directly *ex vivo* and after activation. Importantly, we show that knockdown or blockade of these regulatory receptors enhances the susceptibility of hHSC to killing by oligomerised TRAIL and by TRAIL-expressing NK cells from patients with chronic hepatitis B (CHB). The baseline level of expression of the regulatory receptor TRAIL-R4 dictates the wide variability in susceptibility to TRAIL-induced apoptosis amongst hHSC from different donors, suggesting a role for regulatory TRAIL-Rs in limiting the resolution of liver fibrosis.

## Results

### Primary human hepatic stellate cells express a functional death receptor for TRAIL

**Figure 1:**
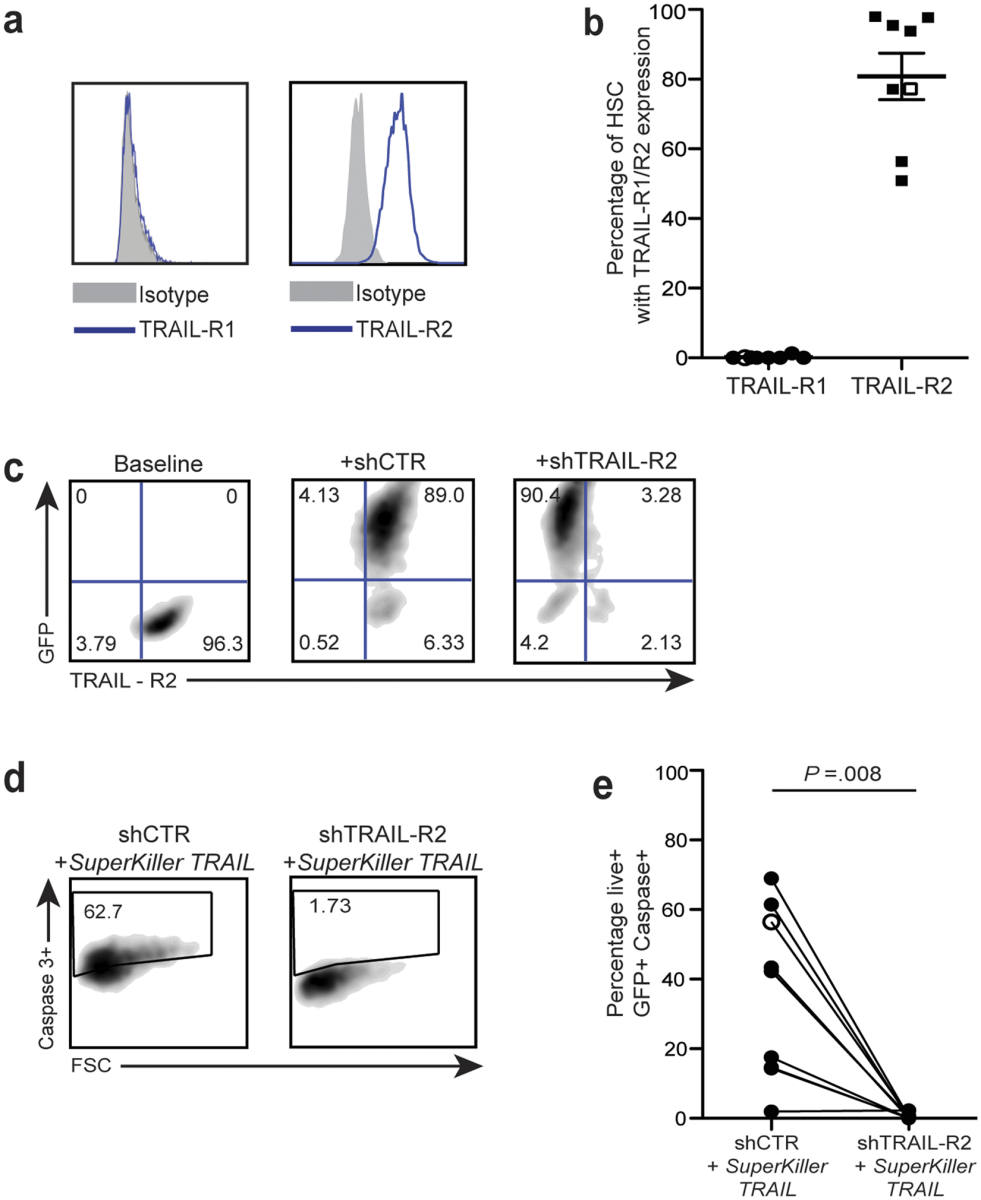
Expression of TRAIL-R2 on primary human HSC has pro-apoptotic function. (**a**) Representative FACS plots of expression level of pro-apoptotic TRAIL-R1 and TRAIL-R2 on hHSC (blue) against matched isotype control (grey). (**b**) Summary of expression levels of TRAIL-R1 and TRAIL-R2 on hHSC from 8 different donors (filled symbols) and LX2 (open symbols). (**c**) Representative FACS plot of levels of TRAIL-R2 at baseline (left), after lentiviral transduction (GFP + ) with shCTR (centre) and shTRAIL-R2 (right). (**d**) hHSC transduced with shCTR and shTRAIL-R2 were treated with *SuperKiller* TRAIL and the degree of apoptosis of transduced cells was assessed by gating on live GFP + cells that were positive for caspase-3. (**e**) Cumulative graph of apoptosis induction by *SuperKiller* TRAIL (% caspase3 + GFP + ) hHSC (filled) and LX2 (open circle) post-knockdown of TRAIL-R2 (shTRAIL-R2) compared to control- short-hairpin RNA (shCTR) transduced hHSC. Baseline levels of caspase-3 post-transduction without addition of *SuperKiller* TRAIL were subtracted.

Primary hHSC were isolated from the healthy margins of liver resections carried out to remove colorectal cancer metastases. After separation on a density gradient, hHSC were cultured and expanded for 2–5 passages to allow transdifferentiation to activated myofibroblast-like cells. The cultured population was uniformly positive for the activated myofibroblast-specific marker anti-smooth muscle actin (αSMA, flow cytometric staining and RT-PCR, Supplementary Figure 1A,B). To investigate activated hHSC susceptibility to killing through engagement with the death ligand TRAIL, cells were stained and analysed by flow cytometry for TRAIL-Rs bearing intracellular death domains able to trigger cell death. Activated hHSC from eight different donors all expressed high levels of the TRAIL death receptor TRAIL-R2, as did the human stellate cell line LX2 (example histograms Fig. 1a, summary data Fig. 1b). By contrast, the alternative death-inducing receptor TRAIL-R1, detected at a much lower level than TRAIL-R2 by RT-PCR and immunoblot of activated hHSC and LX2 in previous studies^13, 26^, was not detectable above isotype staining using flow cytometry (Fig. 1a,b).

To test whether TRAIL-R2 played a functional role on hHSC, we developed shRNA for its selective knockdown. A very high percentage of transduction of hHSC was achieved with lentiviral vectors (mean 90%), as monitored by their expression of GFP (representative example in Fig. 1c). Whilst hHSC transduced with a lentiviral-GFP with an irrelevant shRNA (shCTR) maintained high expression of TRAIL-R2, hHSC transduced with a GFP-expressing lentivirus carrying a shRNA against TRAIL-R2 had significantly reduced mRNA expression and almost completely abrogated protein expression of this death receptor (Fig. 1c and Supplementary Figure 1C).

Primary hHSC were then treated with an oligomerized form of the death ligand TRAIL (*SuperKiller* TRAIL), to simulate the clustering of receptors achieved by cell-bound TRAIL^27^. Induction of stellate cell apoptosis, quantitated by flow cytometry using caspase-3, was compared in live GFP-positive cells transduced with either shCTR or shTRAIL-R2 (representative example, Fig. 1d). *SuperKiller* TRAIL induced a variable degree of apoptosis (mean 36%, range 2–61%) in transduced hHSC from different donors; this was consistently abrogated completely by TRAIL-R2 knockdown (Fig. 1e). These data demonstrated that TRAIL mediates apoptosis of hHSC through TRAIL-R2 and not TRAIL-R1.

### Primary human hepatic stellate cells express the regulatory receptors TRAIL-R3 and TRAIL-R4 directly *ex vivo* and upon activation

**Figure 2:**
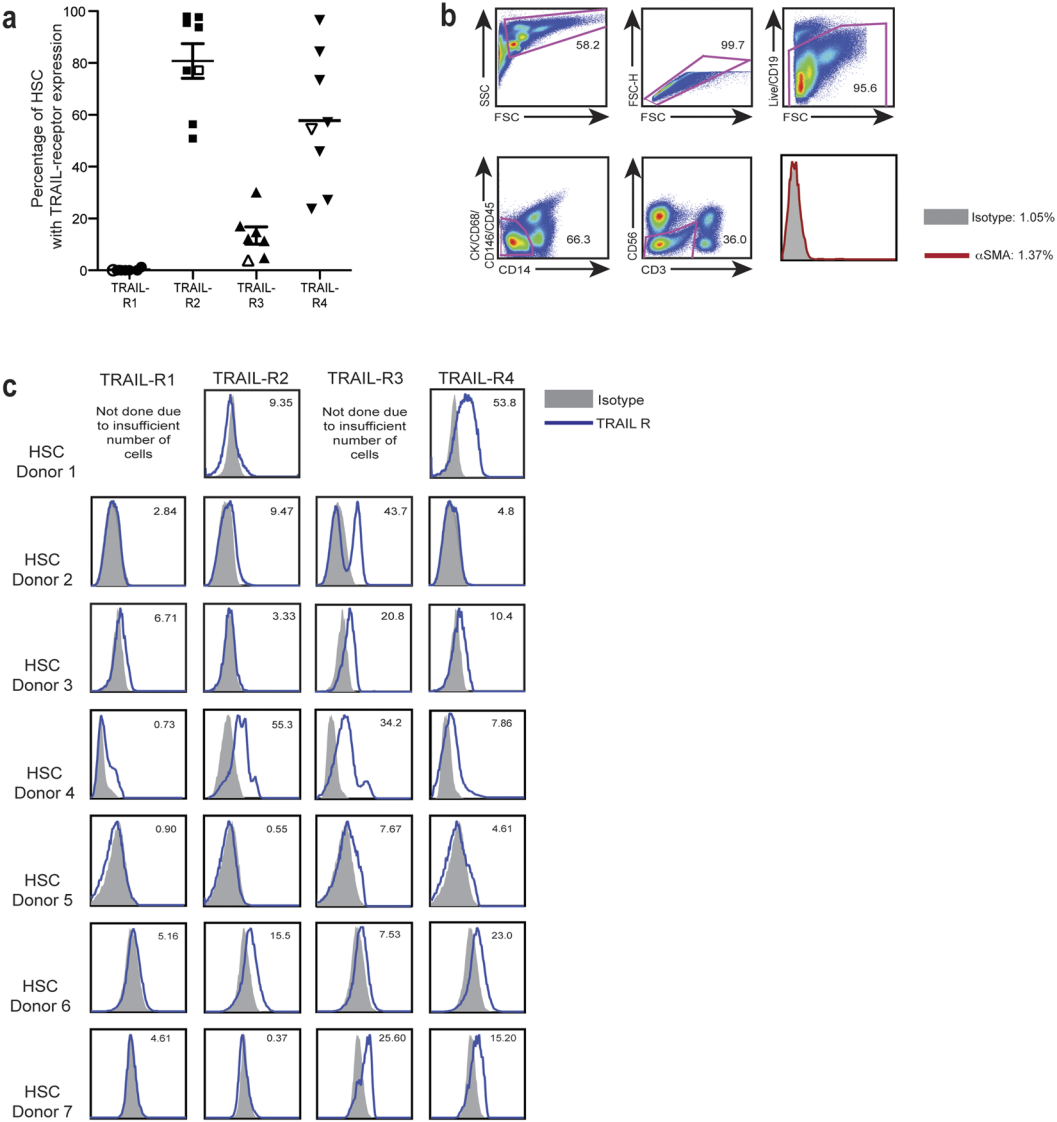
Expression of pro-apoptotic TRAIL-R2 and anti-apoptotic TRAIL-R3 and TRAIL-R4 on hHSC. (**a**) Percentage expression of TRAIL-R1, TRAIL-R2, TRAIL-R3 and TRAIL-R4 on *in vitro* cultured hHSC (filled symbols) and LX2 (open symbols). (**b**) Gating strategy of freshly isolated primary human HSC. All other intrahepatic populations were sequentially gated out (CD19, cytokeratin, CD68, CD146, CD14, CD45, CD3, CD56 shown here excluded from pink gates, + /− CD45 not shown) to identify quiescent hHSC, then confirmed to be negative for αSMA (red line, final histogram). (**c**) *ex vivo* expression of TRAIL-R1–4 on freshly isolated hHSC (blue) against matched isotype control (grey) from seven donors.

hHSC expressing TRAIL-R2 showed a wide-ranging susceptibility to TRAIL-induced apoptosis, raising the possibility of counter-regulation. The receptors TRAIL-R3 and TRAIL-R4 lack intracellular death domains and have been shown to play an inhibitory or decoy role after over-expression in cell lines^18, 22–24, 28–31^. We therefore extended our staining of hHSC (cultured as described above) to include TRAIL-R3 and TRAIL-R4. Activated hHSC not only expressed TRAIL-R2 but also had variable levels of TRAIL-R3 and TRAIL-R4 detectable on their surface (Fig. 2a). The LX2 cell line expressed TRAIL-R4 but not TRAIL-R3 (Fig. 2a).

To determine whether the detection of regulatory receptors on hHSC was an effect of *in vitro* activation, we also stained freshly isolated hHSC for all four surface-expressed TRAIL-Rs. As there are no flow-based markers to identify quiescent HSC, we gated out all other potential parenchymal, non-parenchymal and leucocyte lineages in the liver; the residual quiescent HSC population was negative for αSMA, as expected (Fig. 2b). Direct *ex vivo* TRAIL-R staining was performed on hHSC isolated and stained as above from seven donor livers (Fig. 2c). Variable levels of expression of the four receptors were observed between different donor samples. TRAIL-R1 was expressed at negligible levels, as noted on cultured hHSC. By contrast, staining for TRAIL-R2, R3 and R4 showed clear populations detectable in *ex vivo* hHSC from several donors (Fig. 2c).

### Regulatory receptors limit TRAIL-dependent killing of activated hHSC

**Figure 3:**
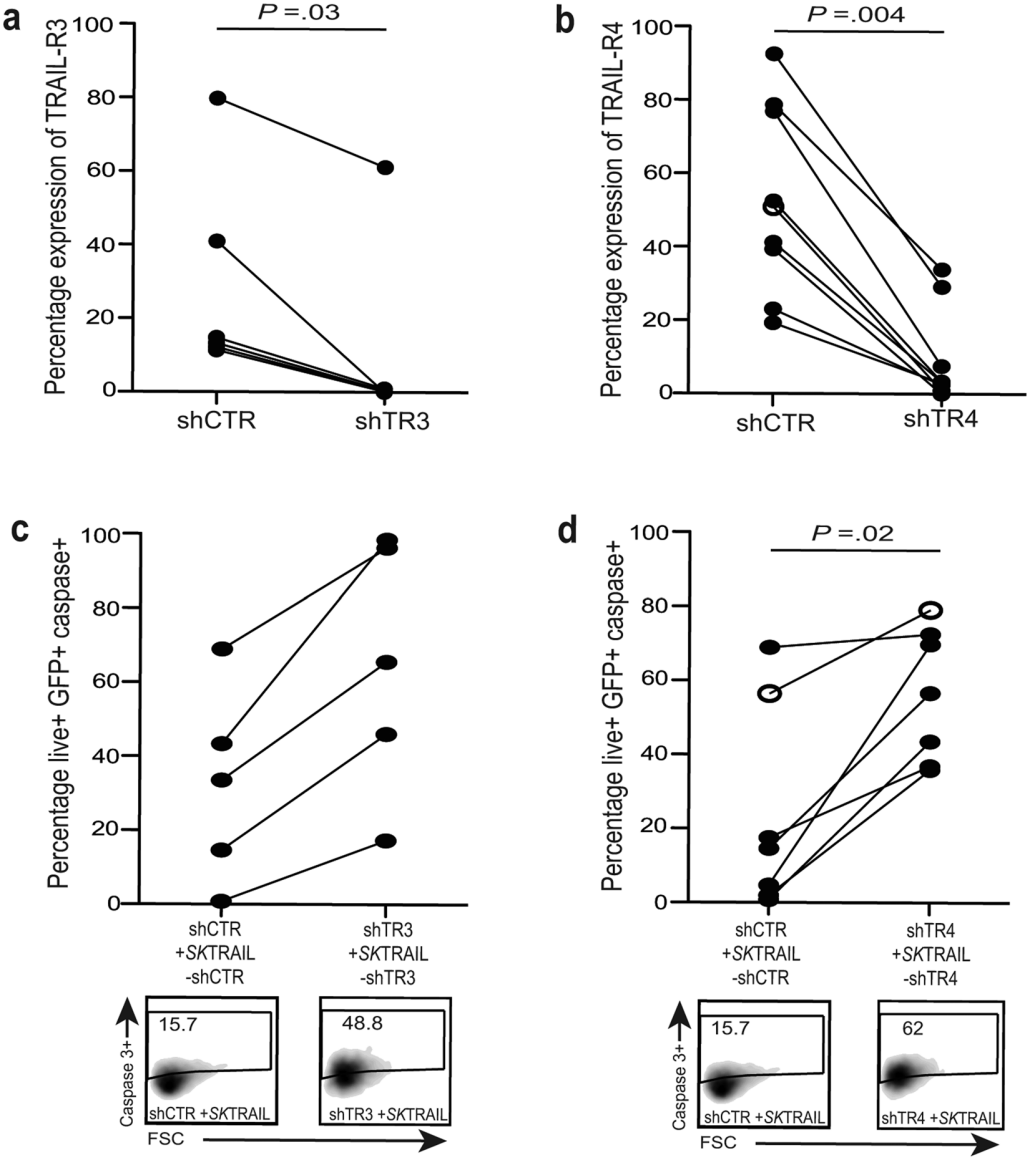
Impact of knockdown of TRAIL-R3/4 on hHSC susceptibility to TRAIL-mediated killing. (**a**) Percentage expression of TRAIL-R3 (left graph) and (**b**) TRAIL-R4 (right graph) on activated hHSC (filled symbols) and LX2 (open symbols) following lentiviral vector transduction with shRNA against TRAIL-R3 or TRAIL-R4 (shTR-3, shTR-4) compared to control short hairpin (shCTR). (**c**) and (**d**) FACS plots and summary data of induction of apoptosis (caspase3+) by *SuperKiller* TRAIL treatment of hHSC transduced with shCTR and shTR-3, shTR-4. Baseline levels of caspase-3 post-transduction without addition of *SuperKiller* TRAIL are subtracted.

We next sought to determine whether the regulatory receptors TRAIL-R3 and TRAIL-R4 expressed on hHSC were capable of partially inhibiting TRAIL-mediated apoptosis. To examine this, we developed shRNA to knockdown TRAIL-R3 or TRAIL-R4. Partial or complete knockdown of these regulatory receptors was achieved following lentiviral transduction of hHSC from different donors with shRNA against TRAIL-R3 and of LX2 and hHSC with shRNA against TRAIL-R4, compared to lentiviruses with control shRNA (Fig. 3a,b). In every donor tested, knockdown of TRAIL-R3 or TRAIL-R4 resulted in enhanced hHSC apoptosis (measured by caspase-3 induction) upon treatment with SuperKiller TRAIL (Fig. 3c,d).

### TRAIL regulatory receptors limit the capacity of NK cells to kill hHSC in CHB

To assess the role of regulatory receptors in stellate cell killing in response to a physiological rather than synthetic TRAIL ligand, we used NK cells from patients with CHB. We have previously found that NK cells from patients with CHB upregulate TRAIL on their surface and are able to kill hepatocytes and HBV-specific T cells through the TRAIL pathway^15–17^. NK cells from patients with hepatitis C have also been found to be able to kill hHSC in a partially TRAIL-dependent manner^12^. NK cells from our cohort of patients with CHB had increased TRAIL compared to healthy controls (Supplementary Figure 2A), in line with our previous studies^15, 17^. Purified NK cells were able to induce variable, mostly limited, degrees of hHSC apoptosis (significantly above baseline apoptosis using NK cells from patients with CHB but not from healthy controls, Supplementary Figure 2B,C). The capacity of NK cells to induce hHSC apoptosis did not correlate with any parameters of CHB disease activity (viral load, ALT, fibrosis score, data not shown).

**Figure 4:**
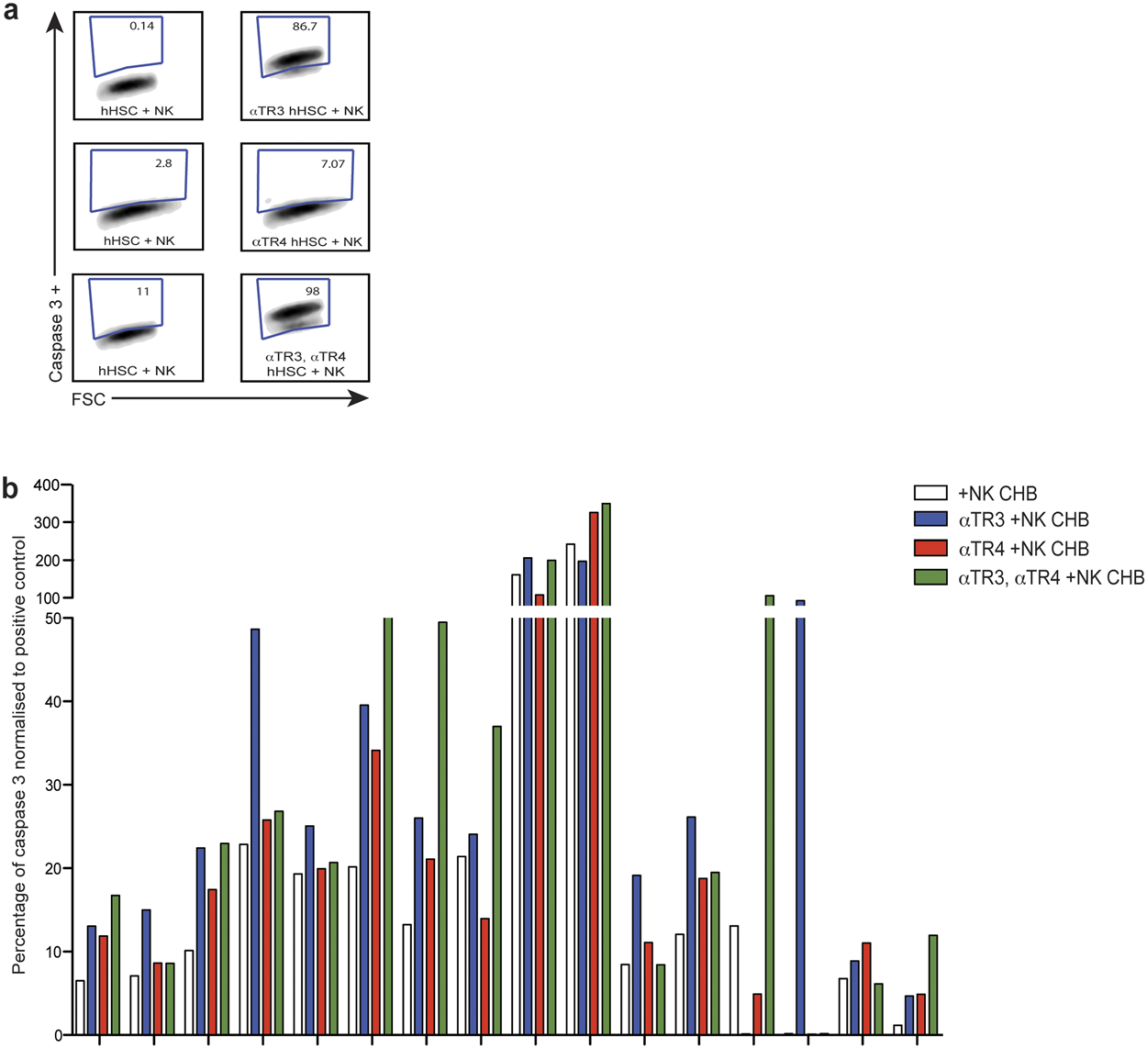
Blocking TRAIL-R3/4 on hHSC increases their susceptibility to NK cell mediated apoptosis. (**a**) FACS plots of induction of apoptosis (caspase3+) in hHSC co-cultured with NK cells from patients with hepatitis B with or without TRAIL-R3 or TRAIL-R4 blockade. (**b**) Percentage apoptosis of hHSC with an isotype blocking mAb (white) or with mAb blocking TRAIL-R3 (αTR3, blue), TRAIL-R4 (αTR4, red) or combination of αTR3 and αTR4 (green bars) upon NK cell co-culture. Responders whose NK cells induced >5% more apoptosis of hHSC with one or more strategy are shown, with %caspase3 induction normalised to the positive control (*SuperKiller* TRAIL) to adjust for inter-donor hHSC variability.

To investigate the role of regulatory TRAIL-Rs in limiting NK cell-mediated killing of hHSC, we took advantage of blocking mAbs that allowed us to scale our assay up to test the large number of samples in our CHB cohort. TRAIL-R3 or TRAIL-R4 blockade or the combination were able to increase the capacity of NK cells to kill hHSC (example FACS plots, Fig. 4a). Using NK cells from a total of 27 patients with CHB, we compared blockade of TRAIL-R3, TRAIL-R4 and the combination (Fig. 4b), showing an enhancement of stellate cell apoptosis in 16 out of 27 patients (59%) with one of more of these approaches. The limiting effects of TRAIL regulatory receptors on stellate cell killing by NK cells from different donors was only partially overlapping and they could therefore act in a complementary, and in some cases synergistic manner (Fig. 4b).

Taken together, these data indicated that blockade of the regulatory receptors TRAIL-R3/4 was able to enhance the induction of stellate cell apoptosis by oligomerised and cell-bound TRAIL ligand.

### Expression of TRAIL-R4 regulates apoptosis of activated hHSC

**Figure 5:**
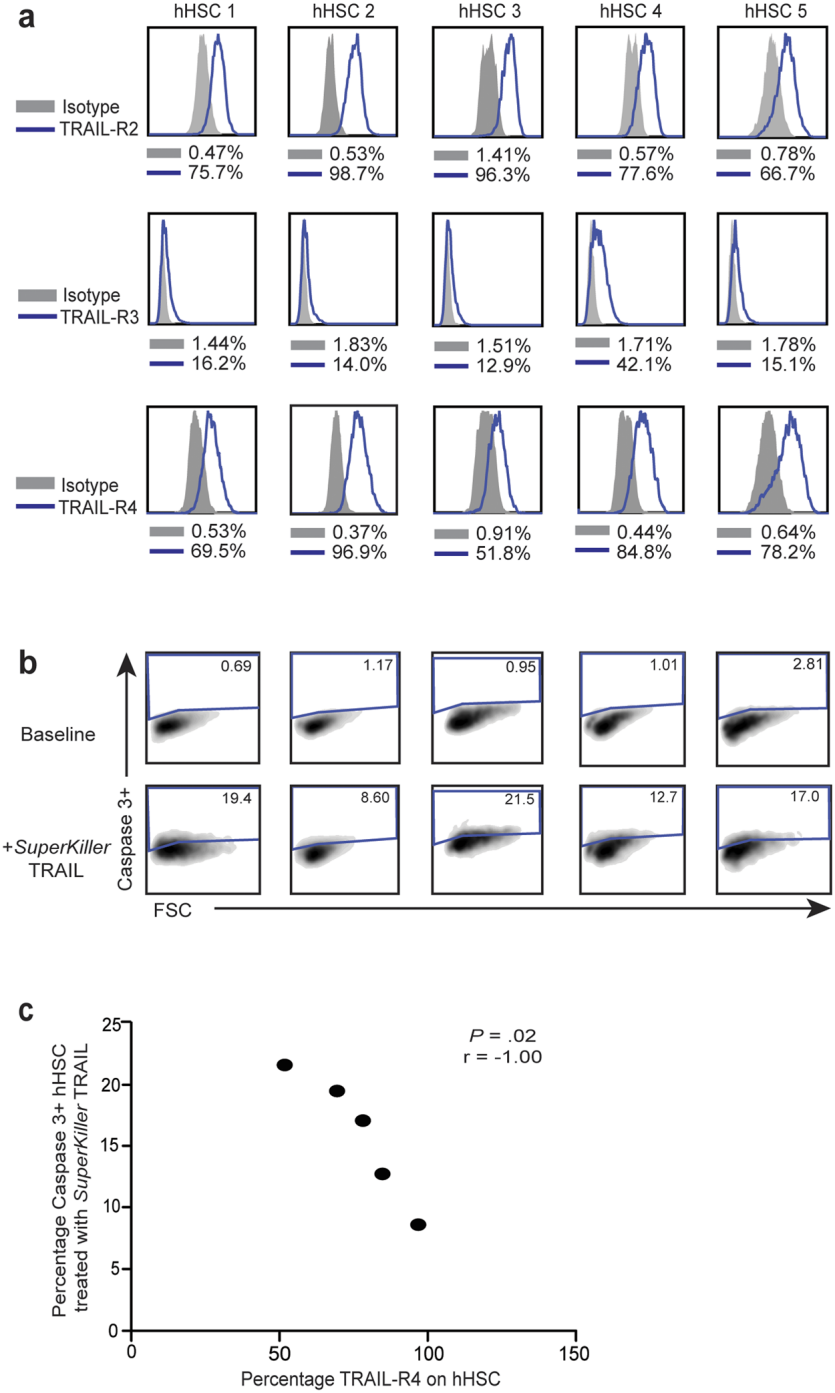
Relationship between expression levels of TRAIL-receptors and resistance to TRAIL-mediated apoptosis. (**a**) Expression levels of TRAIL-R2 (top panel), TRAIL-R3 (middle panel) and TRAIL-R4 (bottom panel) in blue against matched isotype (grey) on *in vitro* culture-activated hHSC from five different donors (hHSC1–5). (**b**) Levels of induction of caspase-3 in hHSC at baseline and on *in vitro* treatment with *SuperKiller* TRAIL. (**c**) Correlation of expression levels TRAIL-R4 on hHSC against their capacity to undergo TRAIL-mediated apoptosis.

Having observed considerable variability in the baseline levels of TRAIL-R2, TRAIL-R3 and TRAIL-R4 on hHSC both *ex vivo* and after activation, we postulated that receptor expression levels may dictate susceptibility to TRAIL-mediated apoptosis. To assess this, we compared the expression of TRAIL-R2, TRAIL-R3 and TRAIL-R4 (as TRAIL-R1 was undetectable) on activated hHSC isolated from liver tissue from 5 different donors (Fig. 5a). Receptor expression levels were then compared with the susceptibility to TRAIL-induced apoptosis of the individual batches of hHSC (Fig. 5b). The level of TRAIL-R2 and TRAIL-R3 did not correlate with induced apoptosis (r = −0.3 P = 0.68 and r = 0.6 P = 0.35 respectively), whereas TRAIL-R4 expression showed a robust inverse correlation with the induction of caspase-3 cleavage in hHSC treated with *SuperKiller TRAIL* (r = −1 p = 0.017, Fig. 5c). This finding suggested that TRAIL-R4 is a critical physiological regulator of hHSC apoptosis.

## Discussion

In order to develop new approaches for the treatment of liver fibrosis, it is essential to gain a better understanding of the factors that can terminate its progression and/or mediate its resolution. A central feature of fibrosis regression is the apoptosis of activated HSC, which is known to be a tightly regulated process^3–5^. A previous study has shown that high levels of the anti-apoptotic protein Bcl-2 in activated hHSC can limit their susceptibility to apoptotic stimuli through the cell-intrinsic pathway^32^. Here we demonstrate a novel mechanism of resistance to cell-extrinsic apoptosis, through variable expression of regulatory receptors for the death ligand TRAIL.

Since the original cloning of the TRAIL-R3 and TRAIL-R4 receptors in 1997^18, 22–25^, there has been debate over their mode of action and limited evidence for a physiological role. Their lack of an intracellular death domain led to the concept that they may act as classical decoy receptors, binding to TRAIL and limiting its engagement of the death-domain-containing receptors TRAIL-R1 and TRAIL-R2^28, 30, 31^. In support of this concept, recent evidence suggests TRAIL-R3/4 may be able to act in both cis and trans, sequestering TRAIL to inhibit killing of neighbouring cells as well as exerting a cell-autonomous effect^33^. Conversely, another study has shown that TRAIL-R4 can inhibit TRAIL-R2 in a non-ligand-dependent manner, involving the formation of TRAIL-R2/4 heterocomplexes^34^. Some have argued that the regulation of TRAIL-induced apoptosis of non-transformed cells is controlled at the level of downstream mediators including c-FLIP, XIAP and Mcl-1 rather than the surface expression of TRAIL-R3/4^28, 35, 36^. Apoptosis regulation mediated by physiological levels of TRAIL-R4 on primary cells has only been demonstrated once, for mitogen-activated T cells^34^ and no physiological inhibitory role for TRAIL-R3 or the combination has been shown before.

Using flow cytometry we were able to show expression of TRAIL-R3 and TRAIL-R4 on both freshly isolated and culture-activated hHSC. This is in line with the recent identification of these inhibitory receptors on stromal cells within tumour tissue^33^. Importantly, our findings revealed novel non-redundant functional roles for both TRAIL-R3 and TRAIL-R4. The fact that shRNA-mediated knockdown of TRAIL-R3/4 was able to sensitise hHSC to apoptosis implied that these receptors can act as primary inhibitors, although it did not exclude additional downstream regulation by mediators such as cFLIP. The pivotal role of TRAIL regulatory receptors in this setting was underscored by the striking correlation between the levels of TRAIL-R4 expressed by hHSC isolated from different livers and their susceptibility to apoptosis induction. Our finding that blockade of TRAIL-R3/4 also enhanced the capacity of NK cells from a number of donors to kill hHSC illustrated that these receptors can exert inhibition of cell-bound TRAIL, even in a setting where there is the potential for it to be overridden by alternative NK cell killing pathways.

Our data highlight the need for further studies to fully elucidate the contribution of TRAIL receptors in determining the lifespan of hHSC and resultant progression versus resolution of liver fibrosis. The capacity we observed of TRAIL-R4, in particular, to calibrate activated hHSC apoptosis *in vitro* suggests it may have merit as a biomarker of the potential for fibrosis progression versus regression. It would be interesting to see whether histological assessment of the balance of expression of death-inducing TRAIL-R2 versus inhibitory TRAIL-R3/4 on hHSC in biopsy tissue from patients with hepatitis B or other liver diseases can serve as a personalised predictor of fibrosis progression. Emerging data emphasise that TRAIL-R2 and TRAIL-R4, in addition to being apoptosis-regulating receptors, have the capacity to drive a pro-inflammatory/pro-proliferative state by activation of NFkB-dependent genes^22, 27, 37, 38^; the possibility that these receptors may therefore actively potentiate liver fibrosis (in addition to limiting its resolution) should therefore be investigated.

Our results also have therapeutic implications. In hepatitis B, we have previously proposed the use of TRAIL blockade as an adjunct to therapeutic vaccination because of its potential to prevent NK cell-mediated deletion of TRAIL-R2-bearing antiviral T cells and hepatocytes and thereby favourably shift the balance of immunity and immunopathology^39^. A concern has been the risk of accelerating liver fibrosis by preventing clearance of activated hHSC by endogenous TRAIL-expressing NK cells; our new data suggest that short-term TRAIL blockade may not have a detrimental effect in patients whose regulatory receptors are already limiting stellate cell apoptosis. Conversely, TRAIL is being considered as an anti-fibrotic therapy in other causes of progressive liver fibrosis due to ongoing liver injury. A recent study showed promising effects of a pegylated form of TRAIL in ameliorating liver cirrhosis by eliminating activated HSC in a rodent model of liver fibrosis^26^. Our findings imply that engagement of TRAIL-R2 without TRAIL-R3/4 would optimise stellate cell killing and the regression of fibrosis. This could be achieved, for example, with TRAIL receptor agonists which have recently been engineered to bind only to TRAIL death-inducing receptors and are unable to engage the inhibitory TRAIL-R3/4^33, 40^. Such an approach is supported by the superior efficacy of a TRAIL-R2 targeted ligand over native TRAIL in eliminating LX-2 or activated rat stellate cells i*n vitro* ^40^; further studies are needed to see if such reagents achieve the predicted enhanced clearance of human HSC.

## Patients and Methods

All experiments were performed in accordance with the approved guidelines and regulations.

### Patient samples

Ethical board approval for the study was submitted through the Royal London Hospital and Royal Free London NHS Foundation Trust. All participants of the study have given written, informed consent. All HBV infected patients were anti-HCV and anti-HIV antibody negative. The patients were stratified on the basis of their disease status (Table 1): viral load (determined by real-time PCR), liver inflammation indicated by serum levels of alanine transaminase (ALT) and degree of fibrosis determined by Ishak stage and/or ELF test (Ishak stage: 1–2 mild fibrosis, 3–4 moderate fibrosis; ELF test: 0–7.7 no/mild fibrosis, 7.7–9.8 moderate to severe fibrosis). For the isolation of primary human HSC, liver tissue was obtained from healthy margins of livers resected for colorectal cancer metastases.

**Table 1:** Clinical characteristics of CHB patients and healthy controls.

	Age in years median (range)	Sex (female:male)	HBeAg+	HBV DNA IU/ml median (range)	ALT IU/L median (range)	Fibrosis (severe/moderate:mild/no)
CHB patients (n = 59)	36 (19–60)	23:26	7/52	1,667 (not detected –100 × 10^6^)	49 (10–748)	43.9:56.09
Healthy Controls (n = 20)	31 (21–35)	9:11	NA	NA	NA	NA

### Isolation of hHSC

Liver tissue was digested using enzymes (1ng/ml of collagenase IV and 0.1ng/ml of DNAse I) and mechanical mashing. Optiprep (Sigma-Aldrich, St Louis, USA) double density gradient (17% and 11.5%) was used to isolate the top layer of retinoid-rich hHSC^41^. The cells were either stained directly *ex vivo* (identity confirmed by autoflourescence and flow cytometric exclusion of other liver cells) or expanded and activated by culture on plastic in SteCM media (ScienCell Research Laboratories, California, USA) as described previously^42^ before being cryopreserved for future experimental use. All hHSC were used for experimental assays between passage 2 and passage 5. The immortalised human HSC cell line LX2, kindly provided by S. Friedman, was cultured in SteCM media and used for experimental assays at passage 2, at a maximum confluency of 80%.

### Isolation of NK cells

NK cells were isolated from whole PBMC by negative selection using an NK cell isolation kit (Miltenyi Biotech, Bergisch Gladbach, Germany) according to the manufacturer’s instructions. The purity of NK cells was checked using flow cytometry by gating on live CD56 + CD3- cells and was always greater than 95%.

### Apoptosis assay

Cryopreserved hHSC were thawed and cultured in T-25 tissue culture flasks (NUNC, Waltham, USA) for 4 days at 37 °C with 5% CO_2_. The hHSC were then detached using 0.05% Trypsin/EDTA (Invitrogen, California, USA) and plated on 48 well plates (Corning, New York, USA). Where LX2 were used in assays, they were treated the same way as pHSC.

Isolated NK cells and hHSC were co-cultured at an E:T ratio of 10:1 for 5 hours in SteCM. After co-incubation, hHSC were harvested and their apoptosis was studied by intracellular staining for active caspase-3 using an active caspase-3 apoptosis kit (BD Bioscience, Oxford, UK) according to manufacturer’s instructions. The data were collected on BD^TM^ LSR II flow cytometer (Beckton Dickinson, New Jersey, USA) and analysed using FlowJo (Treestar, Oregon, USA). The positive control for apoptosis experiments was 500 ng/ml of *SuperKiller* TRAIL (Enzo Lifesciences, Exeter, UK). For these NK-hHSC apoptosis assays, the data has been represented as percentage of caspase 3 normalised to positive control (Fig. 4b, Supplementary Figure 2B). This is done by using the positive control to reflect the maximum (100%) potential of the HSC to undergo apoptosis in each individual experiment, the cleaved caspase 3 levels of HSC at baseline and post co-culture with NK cells are calculated in relation to positive control. This normalisation was done to account for inter-donor variability of hHSC to undergo apoptosis.

### Real-time qPCR

Total cellular RNA was extracted using QIAzol Lysis Reagent and RNeasy Mini Kit (Qiagen, CA, USA) according to the manufacturer’s protocol. After RNA quantification and quality control using NanoDrop1000 System (Thermo Scientific, USA), cDNA was synthesized with MultiScribe reverse transcriptase, random primers, deoxyribose nucleoside triphosphate (dNTP) mix and RNase inhibitor (all Applied Biosystems, CA, USA) according to the following protocol: 2 min 50 °C, 10 min 95 °C, followed by 40 cycles of 15 seconds 95 °C and 60 seconds 60 °C.

6.25 ng of the cDNA sample was used to set up real-time quantitative PCRs reactions using TaqMan® gene expression assays Hs00366278_m1 TNFRSF10B and Hs02758991_g1 GAPDH (Life technologies, CA, USA) and 7500 Fast Real-Time PCR System following the manufacturer’s protocol. Each sample was tested in duplicate. To quantify gene expression, the comparative C_T_ method was used as described previously^43^ using Glyceraldehyde 3-phosphate dehydrogenase (GAPDH) as the internal control. The amplification efficiency of target and reference genes was approximately the same (slope < 0.1).

### Blocking TRAIL-R3/4

TRAIL-R3 (MAB-630, R & D Systems, Minneapolis, USA) and TRAIL-R4 (MAB-633, R & D Systems, Minneapolis, USA) neutralizing monoclonal antibodies (mAb) were used to block these receptors on pHSC. The antibodies were added at a concentration of 5 µg/ml for 30 minutes to HSC cultures. Matched isotypes of these were used as controls. The neutralizing antibodies were washed off using PBS prior to setting up apoptosis assays.

### Surface staining of HSC and NK cells

Freshly isolated hHSC were stained with mAbs against CD19-APC-Cy7, CD146-FITC (BioLegend, London, UK), CD14-v500, CD45-FITC (BD Biosystems, Oxford, UK), CD68-FITC, Cytokeratin-FITC, CD3-PE-Cy7 (eBioscience, Hatfield, UK), CD56-ECD (Beckman Coulter, High Wycombe, UK), TRAIL-R1-PE, TRAIL-R2-PE, TRAIL-R3-PE and TRAIL-R4-PE (R&D Systems, Abingdon, UK) after FcR blocking reagent (Miltenyi Biotec, Surrey, UK) blocking. Appropriate isotype controls were used where necessary. NK cells were stained for CD56-ECD (Beckman Coulter, High Wycombe, UK), CD3-PE-Cy7 (eBioscience, Hatfield, UK), CD16-APC, TRAIL-PE. All cells were stained with Live/Dead® Cell viability stain (Invitrogen, California, USA). Data were acquired on BD^TM^ LSR II flow cytometer (Beckton Dickinson, New Jersey, USA) and analysed using FlowJo (Treestar Oregon, USA).

### Transducing HSC using lentiviral vectors with shRNA

The lentivector pSIN-GFP was digested with EcoRI and BamHI, and the promoter of the gene encoding phosphoglycerate kinase (PGK) was amplified by polymerase chain reaction (PCR) for introduction of EcoRI-BamHI restriction sites. The SFFV promoter from the plasmid pSIN-GFP was replaced with the promoter of the gene encoding PGK, cloned by blunt-end ligation, to generate the plasmid pSIN-PGK-GFP. A second cassette containing the U6 promoter was cloned in this backbone downstream the cPPT sequence between ClaI-EcoRI restriction sites. A BamHI site was introduced downstream of the U6 promoter to clone the short hairpin RNA (shRNA) of interest between BamHI and EcoRI restriction sites. The U6-shRNA cassette was placed upstream the PGK promoter and GFP. All constructs were engineered by standard cloning techniques.

The following shRNA sequences were used:

shTR2: 5′-CTCACTGGAATGACCTCCTTTTTCAAGAGAAAAGGAGGTCATTCCAGTGAG-3′

shTR3: 5′-CTTCCAACAATGAACCTTCTTTTCAAGAGAAAGAAGGTTCATTGTTGGAAG-3′

shTR4: 5′-GATGGTCAAGGTCAGTAATTGTTCAAGAGACAATTACTGACCTTGACCATC-3′

shCTR: 5′-CCTAAGGTTAAGTCGCCCTCGTTCAAGAGACGAGGGCGACTTAACCTTAGG-3′

Transduction was monitored by GFP expression using fluorescence microscopy. Lentiviruses were produced using a packaging cell line (293T-cells) and transfected with p8.91 (encoding structural proteins, gag, pol, rev, tat), pMDG (encoding VSVg env) and transfer vectors pSIN shCTR or pSIN shTR2. The shCTR (shCONTROL), shTR4 (shTRAIL-R4), shTR3 (shTRAIL-R3) and shTR2 (shTRAIL-R2) carrying lentivirus were transduced into HSC plated in a 48 well plate in 500 μl volume of SteCM at an MOI (multiplicity of infection) of 40 for up to three days. Transduction and knockdown was confirmed using FACS.

### Statistical analysis

Wilcoxon signed rank test was performed for all paired data. While comparing non-paired data, Mann-Whitney U test was performed. For correlation data, Spearman’s r test was used.

## Electronic supplementary material

Supplementary Information
