# Supplementary figures and images for: TRAIL regulatory receptors constrain human hepatic stellate cell apoptosis
Source: Sci Rep. 2017 Jul 17;7:5514. doi: 10.1038/s41598-017-05845-5 (PMC5514093; doi:10.1038/s41598-017-05845-5)

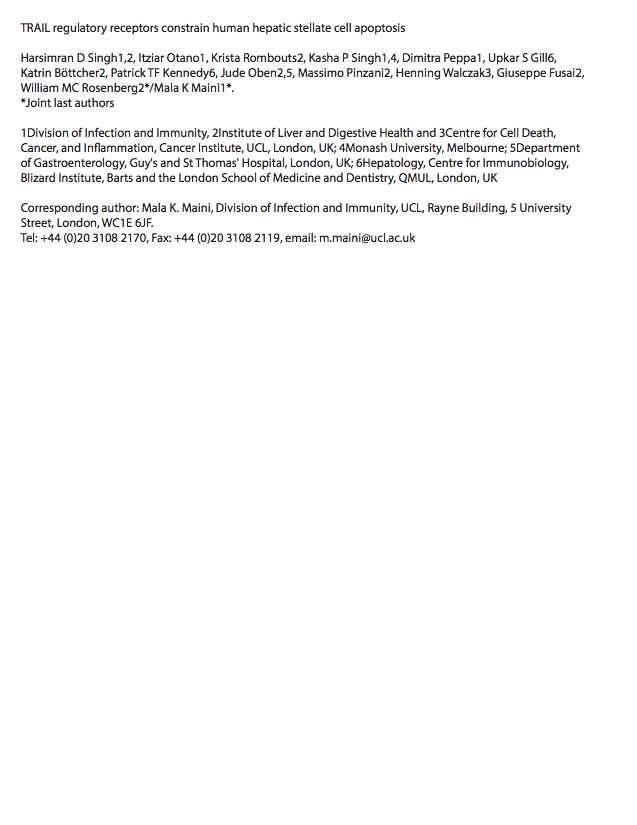

Supplement: Supplementary file 1 — Supplementary Information [file 41598_2017_5845_MOESM1_ESM.tif]
